# The characters of antibodies against PLA2R in healthy individuals and in the patient with PLA2R associated membranous nephropathy

**DOI:** 10.1186/s40001-023-01096-5

**Published:** 2023-03-20

**Authors:** Yan-jiao Cheng, Miao Wang, Jia Wang, Zhao Cui, Ming-hui Zhao

**Affiliations:** 1 Renal Division, Institute of Nephrology, Key Laboratory of Renal Disease, Key Laboratory of CKD Prevention and Treatment, Peking University First Hospital, Peking University, Ministry of Health of China, Ministry of Education of China, Beijing, 100034 People’s Republic of China; 2 Renal Division, Peking University People’s Hospital, Beijing, 100068 People’s Republic of China

**Keywords:** Membranous nephropathy, Phospholipase A2 receptor, Natural antibodies, Antibody titer, IgG subclass, Antibody affinity

## Abstract

**Background:**

Most primary membranous nephropathy (MN) is mediated by anti-phospholipase A2 receptor (PLA2R) antibodies. Recently, these antibodies have been revealed months to years before the disease's onset. Their production and pathogenicity need further investigation.

**Methods:**

Anti-PLA2R antibodies were purified from plasma of eight healthy individuals, 12 patients with PLA2R-related MN and negative circulating antibody (Ab-), and 18 patients with positive anti-PLA2R antibodies (Ab +), using affinity column coupled with recombinant human PLA2R. The antigen specificity, antibody amount, titer, IgG subclass, and affinity were assessed by Western blot, immunofluorescence, ELISA, and surface plasmon resonance.

**Results:**

The natural anti-PLA2R antibodies recognized the conformational structure of PLA2R which locates on the cell membrane of podocytes. The amount of natural IgG was 0.12 ± 0.04 g/L, which accounted for 0.80% of total IgG and was lower than that of patients (2.36%, *P* < 0.001). The titer of natural antibodies was lower than that of patients in Ab- and Ab + groups (1:16 vs. 1:43 vs. 1:274, P < 0.001). IgG2(45.1%) was predominant in natural antibodies, while IgG4 was predominant in Ab + group (45.7 vs. 25.0%, *P* < 0.001). IgG1 was increasing from natural antibodies to Ab- and Ab + groups. The affinity of natural antibodies was lower than that of patients (K_D_: 641.0 vs. 269.0 vs. 99.6 nM, *P* = 0.002). The antibody titer, affinity, and IgG4 percentage were associated with the severity of proteinuria and the stages of membranous lesion.

**Conclusions:**

The natural anti-PLA2R antibodies exist in healthy plasma. The antibody titer, IgG subclass, and affinity may participate in the pathogenesis of anti-PLA2R antibodies.

## Introduction

Primary membranous nephropathy (MN) is a kidney-specific autoimmune disease characterized by the antibodies towards podocyte antigens and the formation of in situ immune complex on the subepithelial area [1]. The major target antigen is M-type phospholipase A2 receptor (PLA2R), with anti-PLA2R antibodies detectable in approximately 70–80% of the patients with primary MN [2].

The pathogenesis of anti-PLA2R antibodies on MN has been demonstrated using the transgenic mice expressing murine full-length PLA2R on podocytes, which can be induced clinical manifestations and pathological tissue manifestations of MN by passive immunization of anti-PLA2R antibodies [3]. In clinical practice, anti-PLA2R antibodies have been demonstrated to be close associations with the severity of proteinuria and the prognosis of kidney function. The higher antibody level is associated with a lower chance of remission achieved spontaneously or after immunosuppressive therapies, and a higher risk of worsening kidney function [4–6]. The change of anti-PLA2R antibodies is parallel or precede the change of clinical manifestations and can predict the disease progression [7–9]. The spreading of epitopes is also associated with a poor prognosis of the disease [10]. However, its occurrence is controversial and the prognosis might be more related to antibody level [11].

The mechanism of anti-PLA2R antibodies production remains unclear. Auto-reactive B cells [12, 13] and natural autoantibodies [14] interacting with self-antigens, such as glomerular basement membrane [15], neutrophil cytoplasmic antigens [16], thyroglobulin [17], insulin [18], and DNA [19], have been reported in healthy individuals. The existence of immunoreactive soluble PLA2R in healthy plasma [20] and the ability of podocytes to secrete vesicles containing PLA2R [21] provide routes for the engagement of PLA2R with the immune system. Circulating PLA2R antibodies have been detectable months to years before the documented proteinuria and biopsy-proven diagnosis in MN patients [22]. Therefore, we hypothesized that the natural antibodies against PLA2R may exist in healthy individuals and the differences between natural antibodies and pathogenic antibodies in MN patients may offer clues for an in-depth understanding of the immune disorders of PLA2R associated MN.

In the current study, we purified anti-PLA2R antibodies by affinity chromatography from the plasma of healthy persons and MN patients, and compared the differences on their immunological features (antibody amount, titer, IgG subclass, and affinity), with the aim to explore the pathogenesis of anti-PLA2R antibodies.

## Materials and methods

### Study population

A total of 30 patients with PLA2R associated MN and eight healthy individuals were enrolled in this study. Kidney biopsy revealed granular deposit of IgG and C3 along glomerular capillary walls on immunofluorescence and the enhanced staining of PLA2R on immunohistochemistry, the glomerular basement membrane thickening on light microscopy, and the electron dense deposit on the subepithelial area on electron microscopy. The causes of secondary MN were excluded, including systemic lupus erythematosus, hepatitis B virus infection, malignancy, medication, heavy metal poisoning, etc.

All the plasma were obtained on diagnosis before the treatments of steroids or immunosuppression, and preserved at − 80 °C. The circulating anti-PLA2R antibodies were detected by a commercial enzyme-linked immunosorbent assay (ELISA) kit (EUROIMMUN AG, Germany). 18 patients were positive of anti-PLA2R antibodies (Ab + group, > 20 RU/ml) and 12 patients were negative (Ab- group, < 14 RU/ml). All the healthy individuals were negative of anti-PLA2R antibodies (HC group, < 2 RU/ml).

The research followed the Declaration of Helsinki and was approved by the ethics committee of Peking University First Hospital. Written informed consent was obtained for sampling tissue and blood.

### The recombinant human PLA2R protein

The extracellular portion of human PLA2R (Acc No.NP_031392.3, amino acid residues 1–1397) was cloned into the HA tag-CMV-14 expression vector and transfected to HEK293 cell. After 48 h, the selection antibiotic G418 (A1720, Sigma, USA) 800 ug/mL was added to establish a stable cell line. To generate the recombinant protein, HEK293 cells were growing in DMEM containing 800 ng/mL G418 and 10% fetal bovine serum (FBS, GIBCO, USA). For harvesting, stable cell lines were cultured in FBS-free DMEM containing 50 µg/mL ascorbic acid (1043003, Sigma) for 5 days. The conditioned medium was collected and precipitated overnight with 3 mol/L (NH4)_2_SO_4_ and centrifuged for 20 min at 14,000 g. The PLA2R protein was redissolved in PBS and incubated with Pierce Anti-HA Magnetic Beads (88837, Thermo Fisher Scientific, USA) for 30 min at room temperature. The bound, HA-tagged proteins were dissociated from the beads using HA peptide (26184, Thermo).

### The purification of anti-PLA2R IgG

The recombinant PLA2R (1 mg/ml) was coupled to the cyanogen bromide activated-Sepharose 4B gel (GE Healthcare, USA) to make a column for affinity purification, with 0.1 mol/l NaHCO_3_ and 0.5 mol/l NaCl, pH 8.3 as the coupling buffer and 0.2 mol/l glycine, pH 8.0 as the blocking buffer.

The IgG fractions from plasma were purified using a protein G affinity column (GE Healthcare). The plasm or IgG fractions were applied to the affinity column coupled with PLA2R, with Tris buffer 0.02 mol/L pH 7.2 for ten column volumes. Anti-PLA2R Antibodies were eluted by glycine buffer 0.1 mol/l containing 0.5 mol/L NaCl pH 2.4 for six column volumes, neutralized with 1 mol/L Tris buffer pH 9.0, and concentrated and exchanged to PBS.

### Western-blot assay

The recombinant PLA2R was electrophoresed with 6.5% polyacrylamide gel at 80 V under non-denatured non-reduced conditions, as well as denatured reduced conditions. The proteins were transferred to a polyvinylidene fluoride (PVDF) paper (Schleicher&Schuell, Germany) by an electrophoretic wet blotting transfer system (GE Healthcare) at 300 mA for 90 min. The PVDF paper was blocked in TBSTM buffer (0.01 mol/l Tris–HCl, pH 7.2, 0.15 mol/l NaCl, 0.1% Tween 20, 30 g/l skimmed milk) for 60 min at room temperature and cut into strips. The strips were incubated with anti-PLA2R Antibodies (Ab + group 1:100, Ab- group 1:4, HC group, 1:4) in TBSTM at 4 ℃ overnight. After three washes, the strips were incubated with HRP-conjugated anti-human IgG (ab6759, Abcam), diluted 1:5000 in TBSTM for 1 h at room temperature. The binding was detected by adding HRP substrate peroxide solution and lumino-image reagent (Millipore, Merck, USA).

### Immunofluorescence assay

The conditionally immortalized human podocytes were kindly provided by Prof. Jochen Reiser (Rush University, Chicago, IL, USA). The protocol of podocyte culturing was described previously[23]. After fixing with 4% paraformaldehyde, the podocytes were blocked with 3% BSA at room temperature for 30 min, and incubated with anti-PLA2R antibodies diluted with 0.01 mol/l PBS, at 4 °C overnight. The slides were then incubated with Alexa Fluor ® 488 affinipure goat anti-human IgG (109-545-003, Jackson, USA), 1:200 at 37 °C for 30 min. The sections were examined on a fluorescent microscope (Nikon, Japan).

### ELISA

The recombinant PLA2R protein (8ug/ml) was coated on half wells of the polystyrene microtiter plates (Nunc, Thermo) in 0.1 mol/l NaHCO_3_, pH 9.6 at 4 °C overnight. The other half was coated with bicarbonate buffer alone to act as antigen-free wells. PBST (0.01 mol/l PBS, 0.1% Tween 20) with 3% BSA was incubated at 37 °C for 1 h to exclude non-specific binding. Anti-PLA2R antibodies were diluted from 1:2 to 1:10240, and incubated at 37 °C for 1 h. The binding was detected by HRP conjugated monoclonal mouse anti-human IgG (Fc specific, ab7499, Abcam, UK) 1:10000 at 37 °C for 30 min. The color was developed for 15 min with TMB (YangGuang BaiQi Biotechnology, Beijing, China), and stopped with 2 mol/l H_2_SO_4_. The results were recorded as the net optical density (OD) (average value of antigen-coated wells minus antigen-free wells) at 450 nm by a microplate reader (Bio-Rad, California, USA). Each plate contained positive, negative, and blank controls. The cut-off value was set as the mean ± 3SD of 20 healthy blood donors (net OD: 0.207). The titer was defined as the highest dilution of the sample which was still positive.

The IgG subclasses were detected by monoclonal mouse anti-human IgG1, IgG2, IgG3 and IgG4 (I5385, I5635, I7260, I7385, Sigma, St. Louis, USA), 1:2500 at 37℃ for 1 h. The binding was detected by alkaline phosphatase conjugated monoclonal goat anti-mouse IgG (A3562, Sigma, St. Louis, USA) 1:5000 at 37℃ for 1 h. The results were recorded as the net OD at 405 nm. The purified anti-PLA2R antibodies from Ab + patients were diluted 1:256, while both the anti-PLA2R antibodies from Ab- patients and healthy individuals were diluted 1:16. The percentage of each subclass was calculated by the OD value of each subclass/the total OD value × 100%.

The IgG concentration was measured by a commercial ELISA kit (ab100547, Abcam, Cambridge, UK) for human IgG detection. The procedure was performed according to the instructions. The plasma was diluted to 1:2 × 10^7^ and the purified antibodies were diluted to 1:2 × 10^5^.

### Measurement of antibody affinity by surface plasmon resonance (SPR)

The carboxymethylated dextran surface of the CM5 sensor chip (GE Healthcare) was activated with a mixture of 0.05 mol/l N-hydroxy succinimide and 0.05 mol/l N-ethyl-N′-[3-diethlyaminopropyl] carbodiimide. The recombinant PLA2R 30 µg/ml in coating buffer (0.01 mol/l sodium acetate, pH 4.5) was immobilized until the desired response units were achieved (1500 RU for Ab + group, 3000 RU for Ab- group, and 8000 RU for HC group). BSA 30 µg/ml in coating buffer was injected to exclude non-specific binding, at 30 µl/min for 300 s, and ethanolamine 1 mol/l (pH 8.5) was injected to block unoccupied activated sites. The purified anti-PLA2R IgG was diluted of eight gradient concentrations in HBS-P (10 mmol/l HEPES, 150 mmol/l NaCl, 0.005% polysorbate 20) and flowed over the immobilized PLA2R proteins at a constant flow rate of 30 µl/min at 25 °C. The antigen–antibody interaction was observed for 180 s of association followed by 360 s of dissociation. The residual antibodies were removed by washing the chip with 10 mmol/l glycine (pH 1.5) for 30 s at 30 µl/min before the next sample. All samples from the same individual were tested on the same plate. The BIA evaluation 4.1 software was used to estimate the K_D_ values.

### Statistical test

All statistical analyses were carried out using SPSS 24.0 (IBM, New York, USA). For data of normal distribution, the results were expressed as mean ± SD. For data of nonnormal distribution, the results were expressed as median (interquartile range). *T*-tests and Mann–Whitney *U* tests were used to assess the differences of quantitative and semiquantitative data. According to distributions, one-way ANOVA or Kruskal–Wallis test was used to compare differences among groups for continuous variables, and the chi-squared test or Fisher exact test was used to compare differences among groups for categorical variables. Pearson or Spearmen correlation was used to analyze the correlations between two variables. The probabilities were two-sided, and P < 0.05 was considered statistically significant.

## Results

### Demographic feature

Of the 30 patients with PLA2R associated MN, 20 patients were males and 10 were females, with a mean age of 51.2 ± 14.7 years. The urinary protein was 3.8 (1.8, 8.5) g/d, serum albumin was 29.2 ± 7.7 g/L, and eGFR was 87.6 ± 24.5 ml/min/1.73m^2^. 18 patients (Ab + group) were positive for anti-PLA2R antibodies with a level of 84.0 (41.8, 221.5) RU/ml. 12 patients (Ab- group) were negative for anti-PLA2R antibodies (< 14 RU/ml), while PLA2R staining was positive along the glomerular capillary walls on kidney biopsy. The urinary protein was significantly higher in the patients with positive antibodies [4.5 (2.7, 8.9) vs. 2.3 (1.5, 3.9) g/d, *P* = 0.025) (Table 1).

**Table 1:** Clinical characteristics of the patients with PLA2R-associated MN

Characters	Anti-PLA2R positive (n = 18)	Anti-PLA2R negative (n = 12)	*P*
Age (years)	53.6 ± 14.5	47.6 ± 14.8	0.282
Gender (male/female)	11/7	9/3	0.694
Clinical features			
Urinary protein (g/24 h)	4.5 (2.7, 8.9)	2.3 (1.5, 3.9)	**0.025**
Serum albumin (g/l)	27.5 ± 7.5	31.9 ± 7.5	0.122
Nephrotic syndrome (n, %)	12 (66.7%)	2 (16.7%)	**0.011**
eGFR (ml/min/1.73m^2^)	83.9 ± 28.9	93.1 ± 15.3	0.269
Anti-PLA2R antibody level (RU/ml)	84.0 (41.8, 221.5)	2.8 (1.0, 6.6)	** < 0.001**
Histological features	n = 15	n = 12	
Stages of membranous injury, I/II/III + IV	7/7/1/0	7/5/0/0	0.500
Intensity of glomerular IgG deposit	3.4 ± 0.4	3.0 ± 0.7	** < 0.150**
Glomerular IgG1/IgG2/IgG3/IgG4 deposit, n	15/7/6/15	12/5/3/12	
C3 deposit, n (%)	13 (86.7)	9 (75)	0.628
Intensity of PLA2R staining	2.7 ± 0.7	2.5 ± 0.6	0.426

*eGFR* calculated by *CKD-EPI* Statistically

significant *P* values are shown in bold

The eight healthy controls (HC group) were three males and five females, with a mean age of 38.3 ± 13.2 years.

### Purification and antigen specificity of anti-PLA2R antibodies

Anti-PLA2R antibodies were purified from the plasma of patients with PLA2R associated MN and healthy controls, by the affinity column coupled with recombinant human PLA2R. Natural anti-PLA2R antibodies could be purified from each of the eight healthy individuals.

**Fig. 1:**
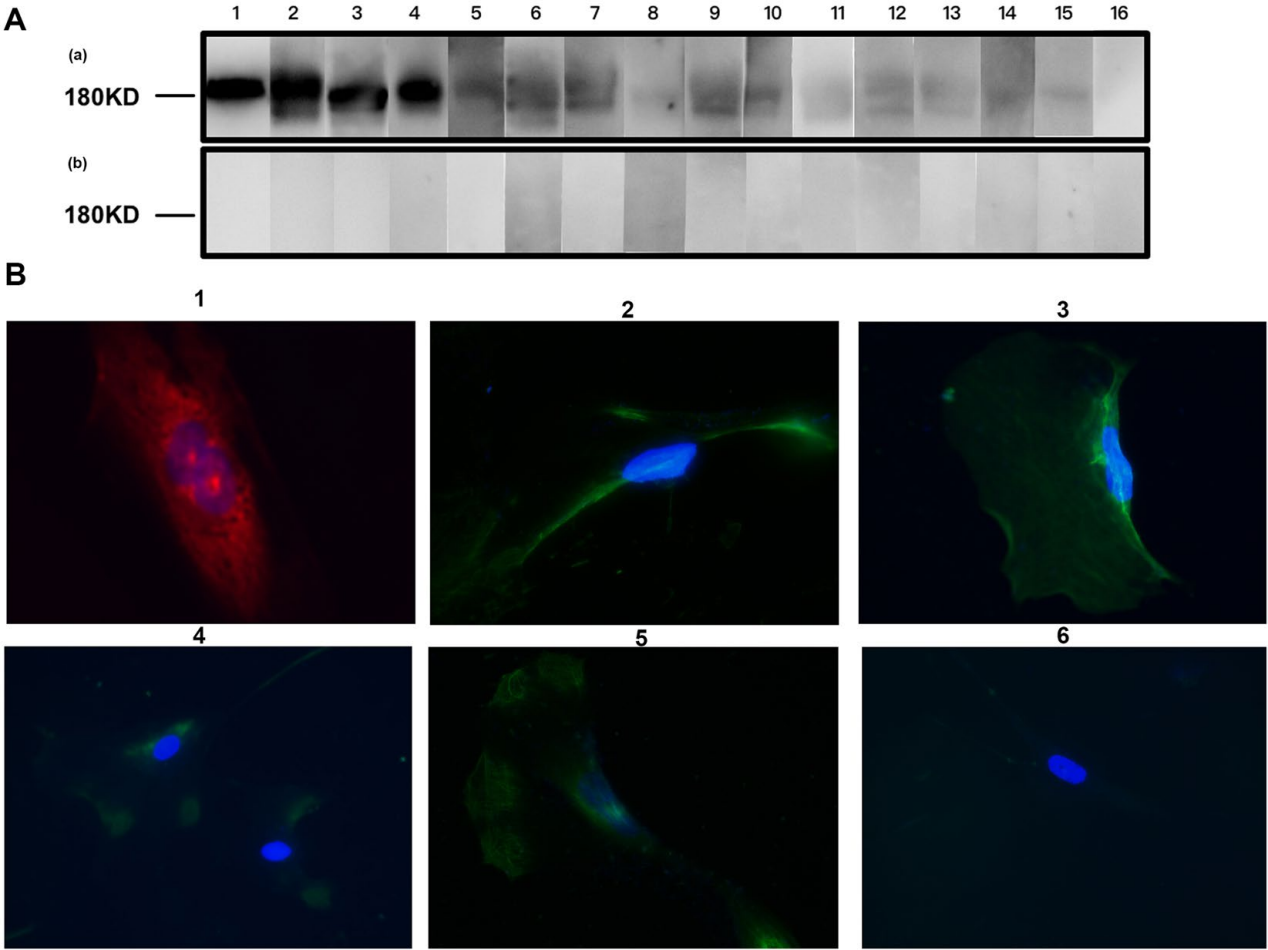
The antigen specificity of anti-PLA2R antibodies. HC group: healthy controls; Ab- group: the PLA2R-MN patients with negative antibody (Ab); Ab + group: the PLA2R-MN patients with positive anti-PLA2R antibodies. **A**. The antigen specificity of purified anti-PLA2R antibodies was detected by Western blot with recombinant human PLA2R as antigen, under nondenatured nonreduced condition (a) or denatured reduced condition (b). Strip 1: positive control (plasma from a patient positive of anti-PLA2R antibodies); strips 2–3: purified anti-PLA2R antibodies from the patients in Ab + group; strips 4–7: purified anti-PLA2R antibodies from the patients in Ab- group; strip 8–15: purified anti-PLA2R antibodies from healthy controls; strip 16: negative control (plasma from a healthy donor). The plasma and purified antibodies from Ab + group were diluted at 1:100, and the purified antibodies from Ab- group and HC group were diluted at 1:4. **B**. The binding of purified anti-PLA2R antibodies to PLA2R on the cell membrane of podocytes was demonstrated by indirect immunofluorescence on human immortalized podocytes. 1. positive control (polyclonal rabbit anti-PLA2R antibodies, 1:200); 2. purified anti-PLA2R antibodies from Ab + group (1:50); 3: purified anti-PLA2R antibodies from Ab- group (1:4); 4–5: purified anti-PLA2R antibodies from HC group (1: 4); 6: negative control (normal human IgG, 1:50)

The antigen specificity of natural anti-PLA2R antibodies was confirmed by Western blot assay using recombinant human PLA2R as a solid-phase antigen. Under the nondenatured nonreduced condition, human PLA2R could be blotted by the natural anti-PLA2R antibodies from all the eight healthy individuals, but not under the denatured reduced condition, as did the anti-PLA2R antibodies purified from the patients in the Ab + group and Ab- group (shown in Fig. 1A).

The binding of natural anti-PLA2R antibodies to PLA2R on podocytes was demonstrated by indirect immunofluorescence on human immortalized podocytes. The natural antibodies could bind to PLA2R on the cell membrane of podocytes, as did the anti-PLA2R antibodies from the patients in the Ab + group and Ab- group (shown in Fig. 1B).

### The IgG amount of anti-PLA2R antibodies

**Fig. 2:**
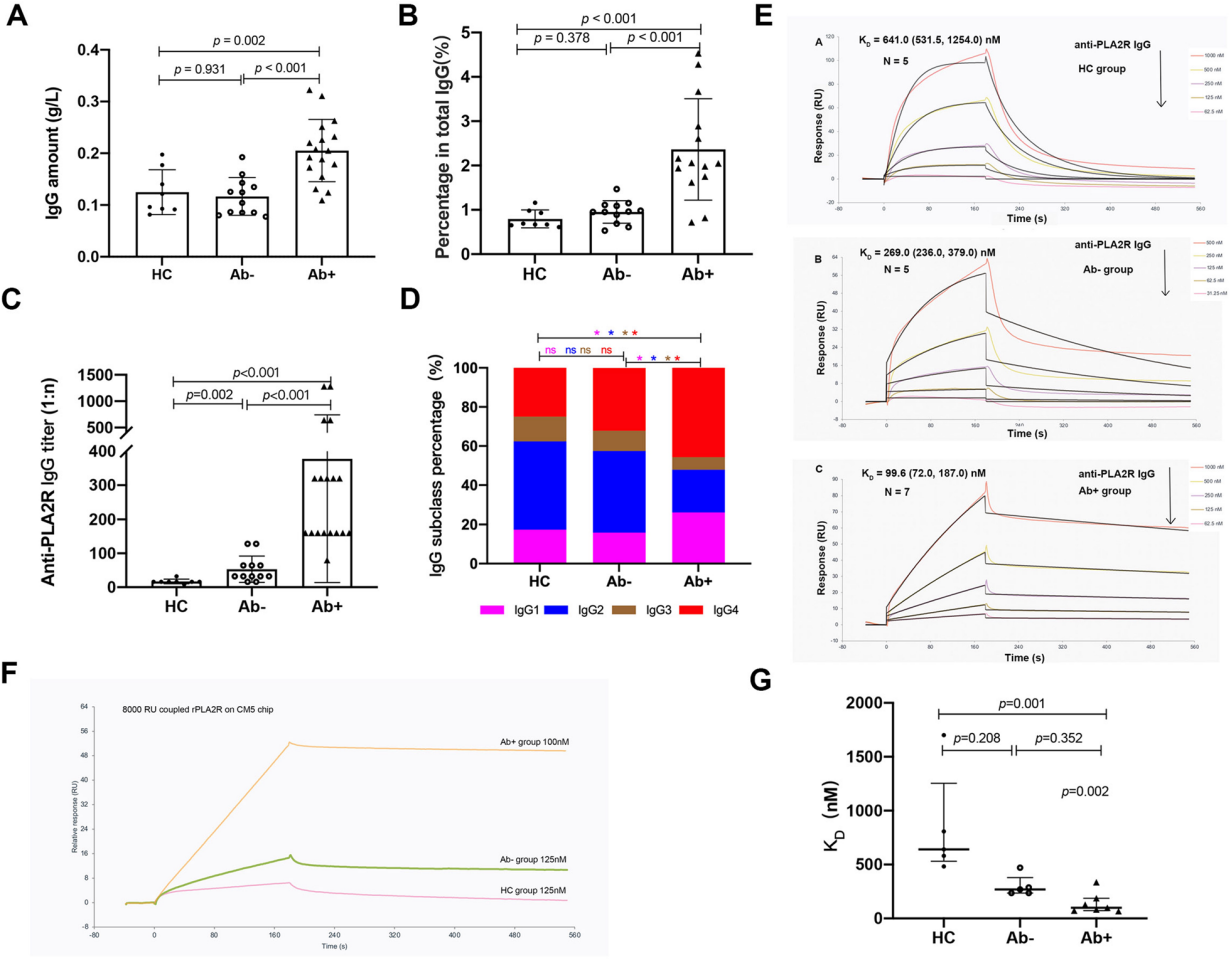
The IgG amount, titer, subclass, and affinity of anti-PLA2R antibodies. HC group: healthy controls; Ab- group: the PLA2R-MN patients with negative antibody (Ab); Ab + group: the PLA2R-MN patients with positive anti-PLA2R antibodies. **A**: The amount of anti-PLA2R IgG in the three groups. **B**: The percentage of anti-PLA2R IgG in total IgG. **C**: The IgG titer of anti-PLA2R antibodies. **D**: The percentage of each IgG subclass in anti-PLA2R antibodies; **E**. The IgG affinity of anti-PLA2R antibodies in the three groups. **F**: The binding of purified anti-PLA2R antibodies to recombinant human PLA2R in SPR. **G**: The comparisons of antibody affinity among the three groups. * *P* < 0.05

In healthy individuals, the amount of natural anti-PLA2R IgG was 0.12 ± 0.04 g/L, which accounted for 0.80 ± 0.20% of total IgG. In the patients of the Ab + group, the amount of anti-PLA2R IgG was 0.21 ± 0.06 g/L, which accounted for 2.36 ± 1.14% of total IgG and was significantly higher than that of natural antibodies (*P* < 0.001). In the patients of the Ab- group, the percentage of anti-PLA2R IgG in total IgG was comparable to that of the HC group (0.95 ± 0.26 vs. 0.80 ± 0.20%, *P* = 0.378) (shown in Fig. 2A, B).

### The IgG titer of anti-PLA2R antibodies

The mean titer of natural anti-PLA2R IgG was 1:16 (log_2_n = 4.00 ± 0.53), including 1:32 in 1/8 individuals, 1:16 in 6/8 individuals, and 1:8 in 1/8 individuals. The mean titer of purified anti-PLA2R IgG was 1:274 (log_2_n = 8.10 ± 1.11) in the Ab + group, which was significantly higher than that of natural antibodies (*P* < 0.001). The mean titer of purified anti-PLA2R IgG was 1: 43 (log_2_n = 5.42 ± 1.00) in the Ab- group, which was also significantly higher than that of natural antibodies (*P* = 0.002) (shown in Fig. 2C).

### The IgG subclasses of anti-PLA2R antibodies

The proportion of each subclass was calculated by its OD value over the total OD value × 100%. Of the natural antibodies from healthy individuals, IgG2 accounted for 45.1 ± 9.3%, followed by IgG4 (25.0 ± 9.6%), IgG1 (17.3 ± 7.2%), and IgG3 (12.6 ± 5.6%) (*P* < 0.001). Of the purified anti-PLA2R antibodies from the Ab + group, IgG4 (45.7 ± 9.3%) was the predominant subclass, followed by IgG1 (26.2 ± 8.0%), IgG2 (21.7 ± 10.8%), and IgG3 (6.4 ± 3.2%) (*P* < 0.001). Of the purified anti-PLA2R antibodies from Ab- group, IgG2 (41.6 ± 8.6%) and IgG4 (32.2 ± 12.0%) (*P* = 0.199) both were the predominant IgG subclasses, followed by IgG1 (15.8 ± 3.4%) and IgG3 (10.4 ± 3.7%) (*P* < 0.001) (shown in Fig. 2D).

Among the three groups, IgG2 was comparable between the HC group and Ab- group (*P* = 0.439) but was higher than that in the Ab + group (*P* < 0.001). IgG4 was the highest in the Ab + group, which was higher than that in Ab- group (*P* = 0.001) and in the HC group (*P* < 0.001). IgG1 was the highest in the Ab + group, compared to Ab-group (*P* < 0.001) and HC group (*P* = 0.004). IgG3 was the least subclass in all three groups (shown in Fig. 2D).

### The IgG affinity of anti-PLA2R antibodies

The affinity of purified anti-PLA2R IgG binding to the recombinant human PLA2R was detected by SPR (shown in Fig. 2E). The binding curves of antibodies showed the lowest maximum response value in the HC group (< 8RU), followed by Ab- group (~ 15 RU), and was much higher in Ab + group (~ 50 RU) (shown in Fig. 2F). The equilibrium dissociation constant (K_D_) of natural anti-PLA2R IgG from healthy individuals was 641.0 (531.5, 1254.0) nM. The K_D_ of anti-PLA2R antibodies from the Ab + group was 99.6 (72.0, 187.0) nM, which was significantly lower than that of natural antibodies (*P* = 0.001), indicating a much higher affinity of anti-PLA2R antibodies in the Ab + group. The K_D_ of anti-PLA2R antibodies from the Ab- group was 269.0 (236.0, 379.0) nM, showing no significant difference from natural antibodies (*P* = 0.208) and that of the Ab + group (*P* = 0.352) (shown in Fig. 2G).

### The correlations among antibody affinity, level, titer, and IgG subclass

**Fig. 3:**
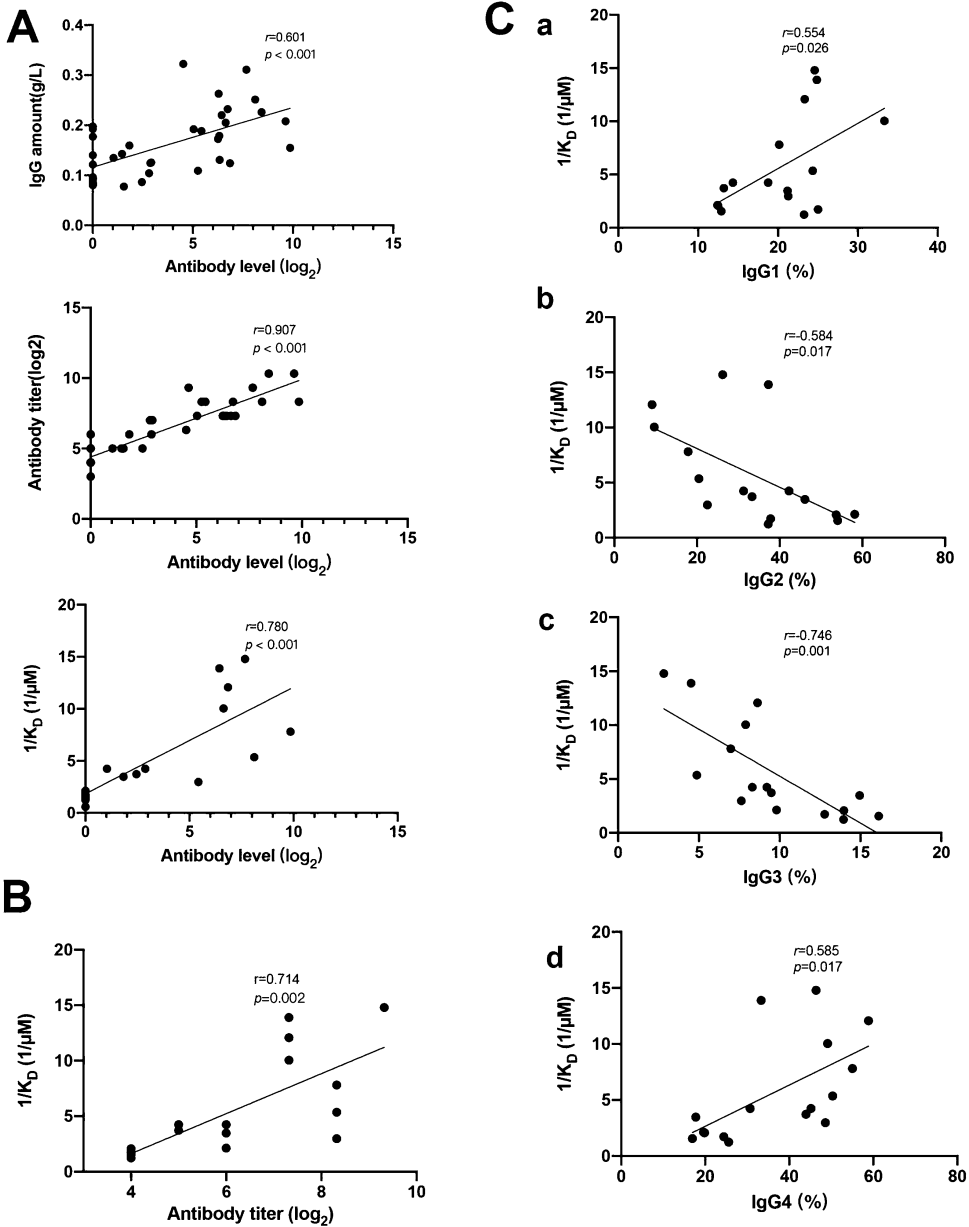
The correlations among anti-PLA2R antibody affinity, level, titer, and IgG subclass. **A**: The correlations between antibody level and antibody amount, antibody titer, and antibody affinity. **B**: The correlations between antibody affinity and antibody titer. **C**: The correlations between antibody affinity and IgG subclass (a: IgG1; b: IgG2; c: IgG3; d: IgG4)

The antibody level of PLA2R antibodies was positively correlated with the antibody amount (*r* = 0.601, *P* < 0.001), antibody titer (*r* = 0.907, *P* < 0.001), and antibody affinity (*r* = 0.780, *P* < 0.001) (shown in Fig. 3A). Partial correlation analysis showed that the antibody level was positively associated with the antibody titer (*r* = 0.644, *P* = 0.018). Linear regression analysis showed that the antibody level was positively associated with antibody titer (*P* < 0.001) and the percentage of IgG4 (*P* = 0.011). The antibody titer was positively correlated with the antibody affinity (*r* = 0.714, *P* = 0.002) (shown in Fig. 3B). The antibody level, titer, and affinity were all positively correlated with the percentage of IgG1 and IgG4, while negatively correlated with the percentage of IgG2 and IgG3 (shown in Fig. 3C).

### The correlation between antibody characteristics and clinical manifestations

**Fig. 4:**
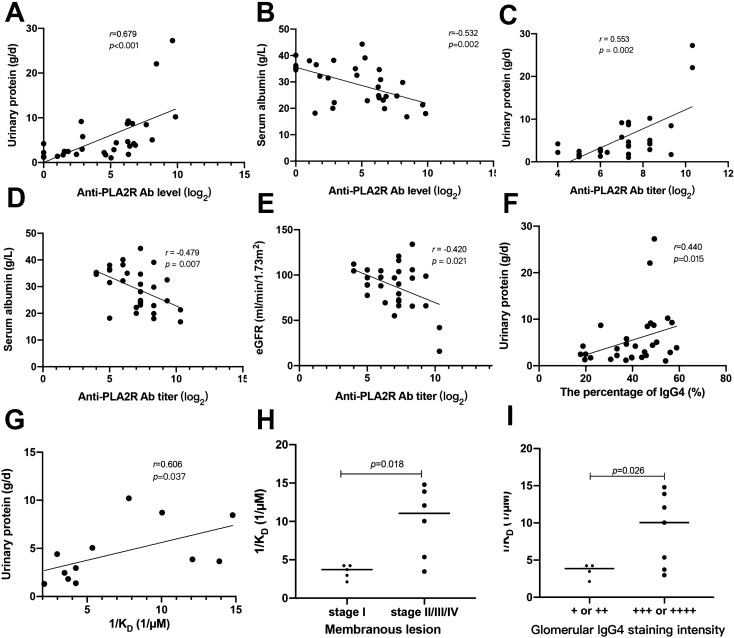
The correlations among anti-PLA2R antibody characters and clinical manifestations. **A**: The correlation between antibody level and urinary protein. B: The correlation between antibody level and serum albumin. **C**: The correlation between antibody titer and urinary protein. **D**: The correlation between antibody titer and serum albumin. **E**: The correlation between antibody titer and eGFR. **F**: The correlation between the percentage of IgG4 and urinary protein. **G**: The correlation between antibody affinity and urinary protein. **H**: The comparison of antibody affinity between the membranous lesion stages. **I**: The comparison of antibody affinity between the intensity of IgG4 staining

The antibody level of anti-PLA2R antibodies was positively correlated with the urinary protein (*r* = 0.679, *P* < 0.001), and negatively correlated with the serum albumin (*r* = − 0.532, *P* = 0.002), but not correlated with eGFR (*P* = 0.276) (shown in Fig. 4A, B).

The antibody titer was positively correlated with the urinary protein (*r* = 0.553, *P* = 0.002), and negatively correlated with the serum albumin (*r* = − 0.479, *P* = 0.007) and eGFR (r = − 0.420, *P* = 0.021) (shown in Fig. 4C–E).

The percentage of IgG4 was positively correlated with the urinary protein (*r* = 0.440, *P* = 0.015), but not correlated with the serum albumin (*P* = 0.158) and eGFR (*P* = 0.919) (shown in Fig. 4F).

The antibody affinity was positively correlated with the urinary protein (*r* = 0.606, *P* = 0.037), but not associated with serum albumin (*P* = 0.104) or eGFR (*P* = 0.698) (shown in Fig. 4G). The patients with the membranous lesion stage II and above presented with higher antibody affinity, compared to those with stage I (*P* = 0.018) (shown in Fig. 4H). The patients with stronger intensity of IgG4 staining presented with higher antibody affinity of anti-PLA2R antibodies (*P* = 0.026) (shown in Fig. 4I).

## Discussion

In the current study, we found the existence of natural antibodies against PLA2R in the healthy plasma, which could be purified by affinity chromatography. The IgG amount, antibody titer, and affinity of them were significantly lower than those of the pathogenic anti-PLA2R antibodies from MN patients. The IgG subclass distribution was IgG2 predominant in the natural antibodies, while it was IgG4 predominant in MN patients. These differences in the immune features may participate in the mechanism of PLA2R antibodies mediated MN. The target antigen of natural antibodies was the conformational structure of PLA2R, which is the same as that of the pathogenic antibodies; however, its fine specificity needs further investigations.

PLA2R locates on the surface of many cells in a wide variety of tissues [24]. The recombinant soluble PLA2R suppresses the integrin β1-mediated podocyte migratory response to collagens [20]. Immunoreactive PLA2R has been detected in human plasma. Thus, it is expected that humans must have established immune tolerance to this antigen [25]. In this study, we purified the natural antibodies against PLA2R from the plasma of all eight healthy individuals. Their IgG amount, antibody titer, affinity, and IgG subclasses were all consistent in every subject, suggesting that these natural antibodies may generally exist in normal persons and participate in the immune homeostasis to PLA2R.

In contrast to the commonly reported IgM type of natural antibodies, which compose an anti-unique network for maintaining immune tolerance by binding to F(ab')2 fragments of autologous IgG and inhibiting its binding to self-antigens[26], the IgG type of natural antibodies usually present with some potentially pathogenic features. Burbelo PD, et al. [22] have reported the anti-PLA2R IgG detected months to years before the full onset of MN and kidney biopsy diagnosis, suggesting the existence of non-pathogenic or less-pathogenic anti-PLA2R antibodies, thus the changing of antibody pathogenicity may be associated with the disease onset.

In this study, we found that the amount of natural anti-PLA2R IgG and its percentage in total IgG were significantly lower than those of MN patients. The titer of natural anti-PLA2R antibodies was remarkably low as well, suggesting that the increased quantity of antibodies may be an important feature of its pathogenicity. Studies have shown the natural antibodies to be pathogenic only when a threshold is exceeded [27, 28]. The levels of natural IgG are fluctuated, presumably related to exposure to antigens [29]. A close association has been revealed between the elevated air pollution index PM2.5 and the increased prevalence of MN in the last two decades in China [30]. The long-term exposure to PM2.5 promotes the production of autoantibodies and immune complexes [31]. The air pollution increases the circulating levels of inflammation mediators such as TNF-α and IL-6 [32, 33]. The expression of PLA2R in type II alveolar epithelial cells was markedly enhanced after the endotoxin challenge in mice where a remarkable elevation of TNF-α has been observed. The circulating level of soluble PLA2R was also significantly elevated after the exposure to endotoxin [34]. These findings imply that the increase of PLA2R antigens after exposure to air pollution may induce an elevated level of anti-PLA2R antibodies and the development of MN. Besides the difference in antibody level between the natural antibodies and pathogenic ones, we also found close associations between the antibody level/titer and the clinical features such as urinary protein, serum albumin, and serum creatinine in MN patients. These results are consistent with many previous studies [4–9] and highlight the importance of antibody amount in its pathogenicity.

The IgG subclass switch was observed between the predominance of IgG2 in natural anti-PLA2R antibodies and the predominance of IgG4 in MN patients. Recently, Haddad G, et al. [35]. have demonstrated a mechanism by which the aberrantly glycosylated anti-PLA2R IgG4 directly bond mannose-binding lectin in a glycosylation-dependent manner, activate the lectin pathway of complement activation, induced proteolysis of the essential podocyte proteins synaptopodin and NEPH1 and result in podocyte injury in MN. IgG4 is the last one to appear during the process of IgG subclass switch, which has the highest affinity to the antigens and implies long-term exposure to the antigens. In this study, we found positive correlations of the antibody affinity with the percentage of IgG4 and the intensity of glomerular IgG4 staining. These findings highlight the importance of the IgG subclass switch to IgG4 for the anti-PLA2R antibodies to be pathogenic. We also found that the percentage of IgG1 was increasing from it in natural antibodies to that in pathogenic ones. IgG1 has been reported to precede IgG4 deposition in stage I of MN, whereas IgG4 is predominant in later stages [36]. IgG1 and IgG3 deposits have been revealed in pediatric patients with segmental MN, whereas IgG1-4 were detected in those with global MN [37]. Therefore, the raise of IgG1 may also participate in the pathogenesis of anti-PLA2R antibodies.

In the current study, we found that the affinity of natural anti-PLA2R antibodies was remarkably low, while it was higher in the MN patients with undetectable antibodies and it was highest in the MN patients with positive anti-PLA2R antibodies. This suggests an affinity maturation process during the development of MN. In MN patients, the antibody affinity was positively correlated with the urinary protein. The patients with membranous lesion stage II and above presented with higher antibody affinity. These findings imply the involvement of antibody affinity in the pathogenesis of anti-PLA2R antibodies.

The antigen of natural anti-PLA2R antibodies was examined as the non-denatured non-reduced conformational structure of PLA2R which locates on the cell membrane of podocytes, the same as the antigen recognized by anti-PLA2R antibodies from MN patients. The major B-cell epitopes of anti-PLA2R antibodies have been defined in the CysR domain, in addition to that, other epitopes are also reported in CTLD1, CTLD7, and possibly CTLD8 domains [11, 38, 39]. The fine epitopes of natural antibodies need to be further investigated.

In the present study, we found that the immune characters of anti-PLA2R antibodies were changing among the natural antibodies, the antibodies of 2-14U/ml, and the antibodies over 14U/ml. The antibody level was positively correlated with the antibody amount, titer, and affinity. These findings imply that although the cut-off value of anti-PLA2R antibodies has been set as 14 U/ml for diagnosis with high specificity, the results of anti-PLA2R antibodies under 14 U/ml are still of clinical value, especially those close to 14 U/ml or increasing gradually, which may imply a mild pathogenic role of anti-PLA2R antibodies.

In conclusion, the natural anti-PLA2R antibodies exist in healthy human plasma and could be purified by affinity chromatography. The differences between natural antibodies and pathogenic antibodies from MN patients indicate that the higher antibody amount, IgG4 subclass switch, and affinity maturation are involved in the pathogenesis of anti-PLA2R antibodies mediated MN.

## Data Availability

All data generated or analyzed during this study are included in this article. Further enquiries can be directed to the corresponding author.
