# Correction: The characters of antibodies against PLA2R in healthy individuals and in the patient with PLA2R associated membranous nephropathy

**DOI:** 10.1186/s40001-023-01110-w

**Published:** 2023-04-05

**Authors:** Yan-jiao Cheng, Miao Wang, Jia Wang, Zhao Cui, Ming-hui Zhao

**Affiliations:** 1 Renal Division, Institute of Nephrology, Key Laboratory of Renal Disease, Key Laboratory of CKD Prevention and Treatment, Peking University First Hospital, Peking University, Ministry of Health of China, Ministry of Education of China, Beijing, 100034 People’s Republic of China; 2 Renal Division, Peking University People’s Hospital, Beijing, 100068 People’s Republic of China

**Correction: European Journal of Medical Research (2023) 28:128** 10.1186/s40001-023-01096-5

In the original publication of the article [1], the *P* value of “Intensity of glomerular IgG deposit” in Table 1 was incorrectly published as **< 0.150** instead of 0.150. The original article has been corrected.
